# Total Arch and Descending Aorta Replacement for Retrograde Type A Aortic Dissection After Endovascular Stent Graft Replacement for Complicated Type B Aortic Dissection

**DOI:** 10.7759/cureus.5017

**Published:** 2019-06-27

**Authors:** Kazuyuki Ishibashi, Mamika Motokawa

**Affiliations:** 1 Cardiovascular Surgery, Yonemori Hospital, Kagoshima, JPN

**Keywords:** endovascular surgery, aortic dissection, tevar

## Abstract

Retrograde type A aortic dissection (RTAD) is a severe complication of thoracic endovascular aortic repair (TEVAR). In this regard, we present our unique surgical methods for total arch and descending aorta replacement for RTAD after TEVAR for complicated type B aortic dissection (TBAD). A 52-year-old man with a history of distal arch large aneurysm was diagnosed with TBAD. Because he had sustained chest pain and his aneurysm diameter was 67 mm, TEVAR was urgently performed. After a right axillary-left axillary artery bypass, a stent graft was deployed in the descending aorta via the right femoral artery. Coil embolization was performed in the left subclavian artery. After the condition of the stent graft was checked by angiography, no Type 1 endoleak and backflow from the re-entry was observed. However, seven days after the operation, he experienced chest pain suddenly.

Computed tomography (CT) revealed forward blood flow in the descending aorta (type IA endoleak) and thrombosed aortic dissection in the ascending aorta. The distal arch diameter exceeded 70 mm. A decision was taken to immediately perform an operation. Total aortic arch and descending aorta replacement were performed through a median sternotomy with left 5th interspace thoracotomy. The operation was performed under deep hypothermic circulatory arrest, and selective antegrade cerebral perfusion was accomplished. As a result of the exploration of the aortic arch, it was found that the intimal injury by the bare stent caused RTAD. The patient was successfully extubated after the operation and was discharged without any complications. RTAD can present as an early complication after descending stent grafting because of aortic instability or due to the strength of bare stents. Aortic arch and descending aorta replacement after TEVAR via a clamshell incision can be safely performed if RTAD is diagnosed early.

## Introduction

Thoracic endovascular aortic repair (TEVAR) has been increasingly performed for treating a complicated type B aortic dissection (TBAD). TEVAR is a less invasive procedure and is associated with lower mortality and fewer complications than traditional open surgery [1-3]. However, one of the most severe complications of this procedure is retrograde type A aortic dissection (RTAD), which has a low incidence but a high mortality rate [4-7]. Ascending aorta and total arch replacement via median sternotomy has been generally performed for RTAD. Furthermore, after TEVAR for complicated TBAD, re-intervention may be necessary due to endoleak or expanding patent false lumen of the descending aorta by a large residual entry [8-11]. Here, we report a new method for the total arch and descending aorta replacement for RTAD after TEVAR for complicated TBAD using which we succeeded in the exclusion of the false lumen of the descending aorta.

## Case presentation

**Figure 1:**
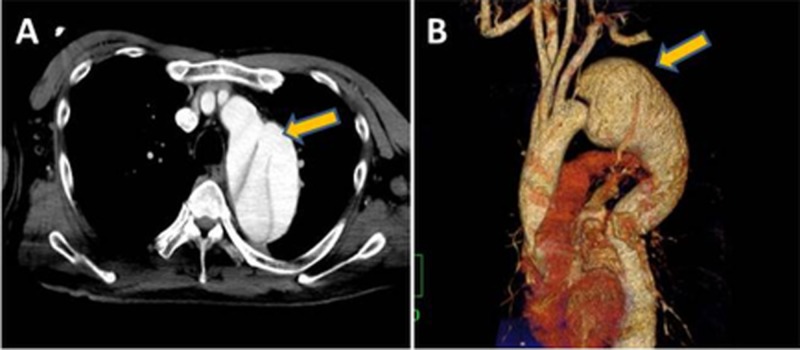
A-B. Preoperative computed tomography image: Enlargement of type B aortic dissection to 65 mm with a primary tear distal to the left subclavian artery

**Figure 2:**
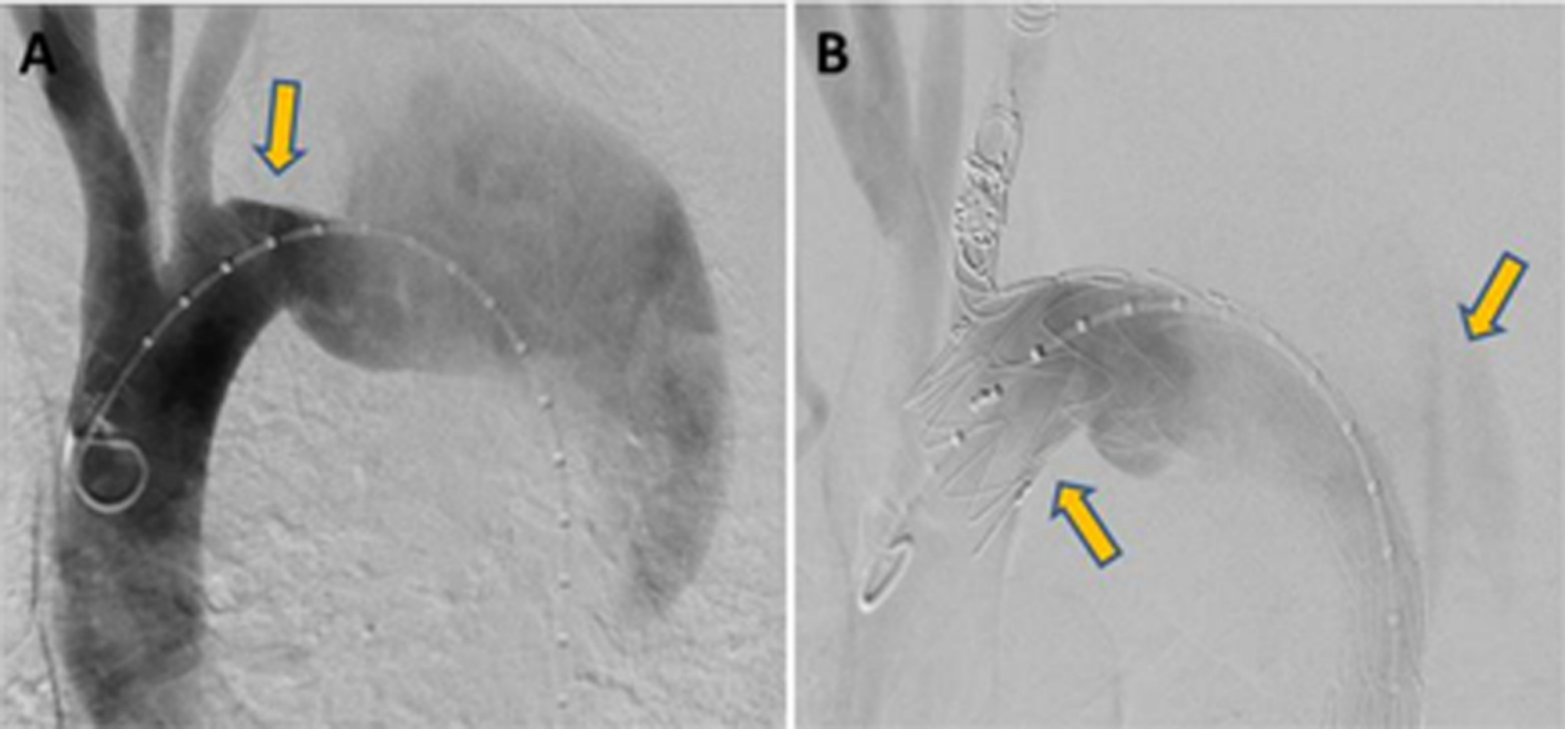
Angiogram showed: (A) There was a 30-mm landing zone between the left common carotid artery and the primary tear (aneurysm) (B) After thoracic endovascular aortic repair for type B aortic dissection.

 A 52-year-old man with a history of a distal arch aneurysm was admitted because of complicated acute TBAD. Contrast-enhanced computed tomography (CT) showed that the proximal thoracic aorta diameter was 67 mm, and proximal entry was found distal to the left subclavian artery (LSA) (Figure 1). His chest pain progressively worsened despite an antihypertensive being administered. Therefore, there was a likelihood of impending rupture, and TEVAR with concomitant LSA revascularization was urgently performed. After right axillary-left axillary artery bypass with a 6-mm-ringed artificial PROPATEN vascular graft (W. L. Gore & Associates, Inc., Flagstaff, USA), a 32×32×150-mm Variant Captiva stent graft (VAMF3232150, Medtronic, MN, USA) was deployed in the descending aorta in Zone 2 via the right femoral artery. Subsequent coil embolization of the LSA stump with a Tornado Embolization Coil (Cook Medical, Blooming, IN, USA) was performed to avoid Type II endoleaks. A 3-cm landing zone was present proximally (Figure 2). We then checked the condition of the stent graft by angiography; the entry tear exclusion was confirmed, and no Type I endoleak and backflow from the distal re-entry was observed (Figure 2). Balloon dilatation of the stent graft was not performed.

**Figure 3:**
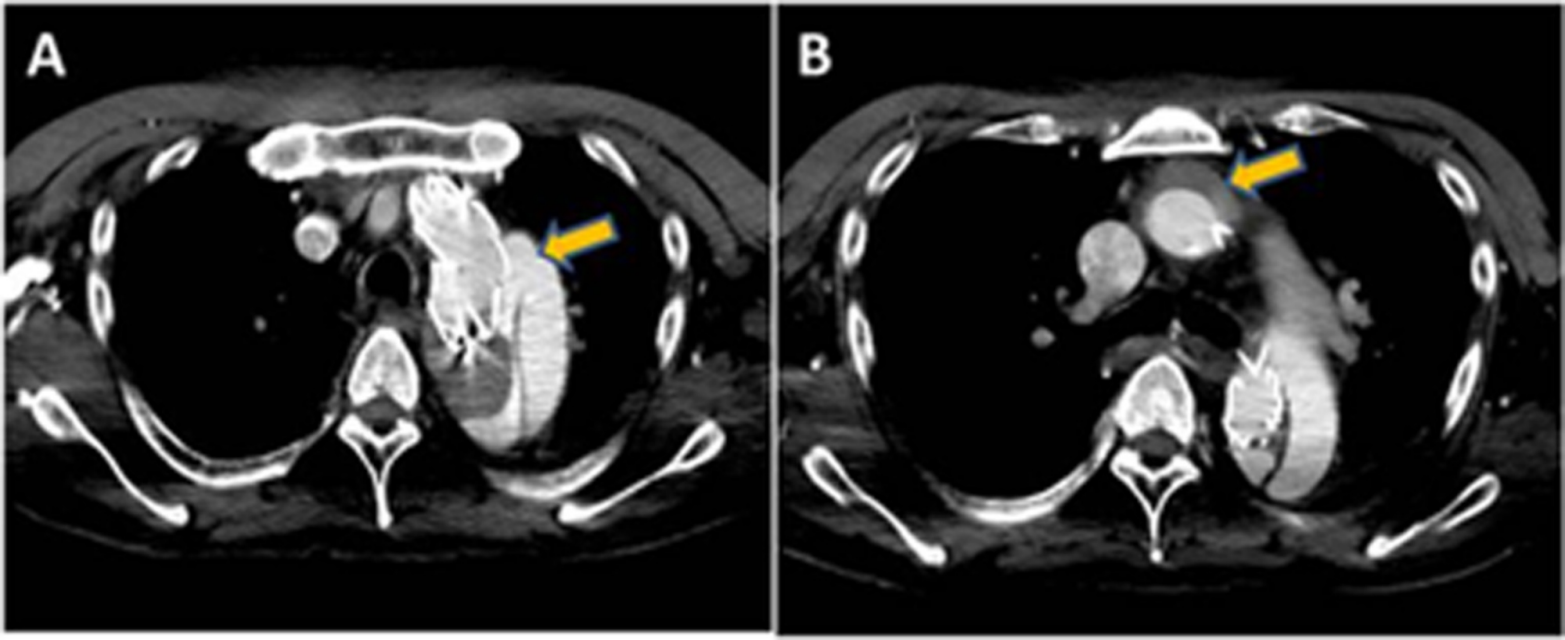
Computed tomography findings: The enlarged patent false lumen of the descending aorta with a primary entry (A) and thrombosis of the ascending aorta (B)

**Figure 4:**
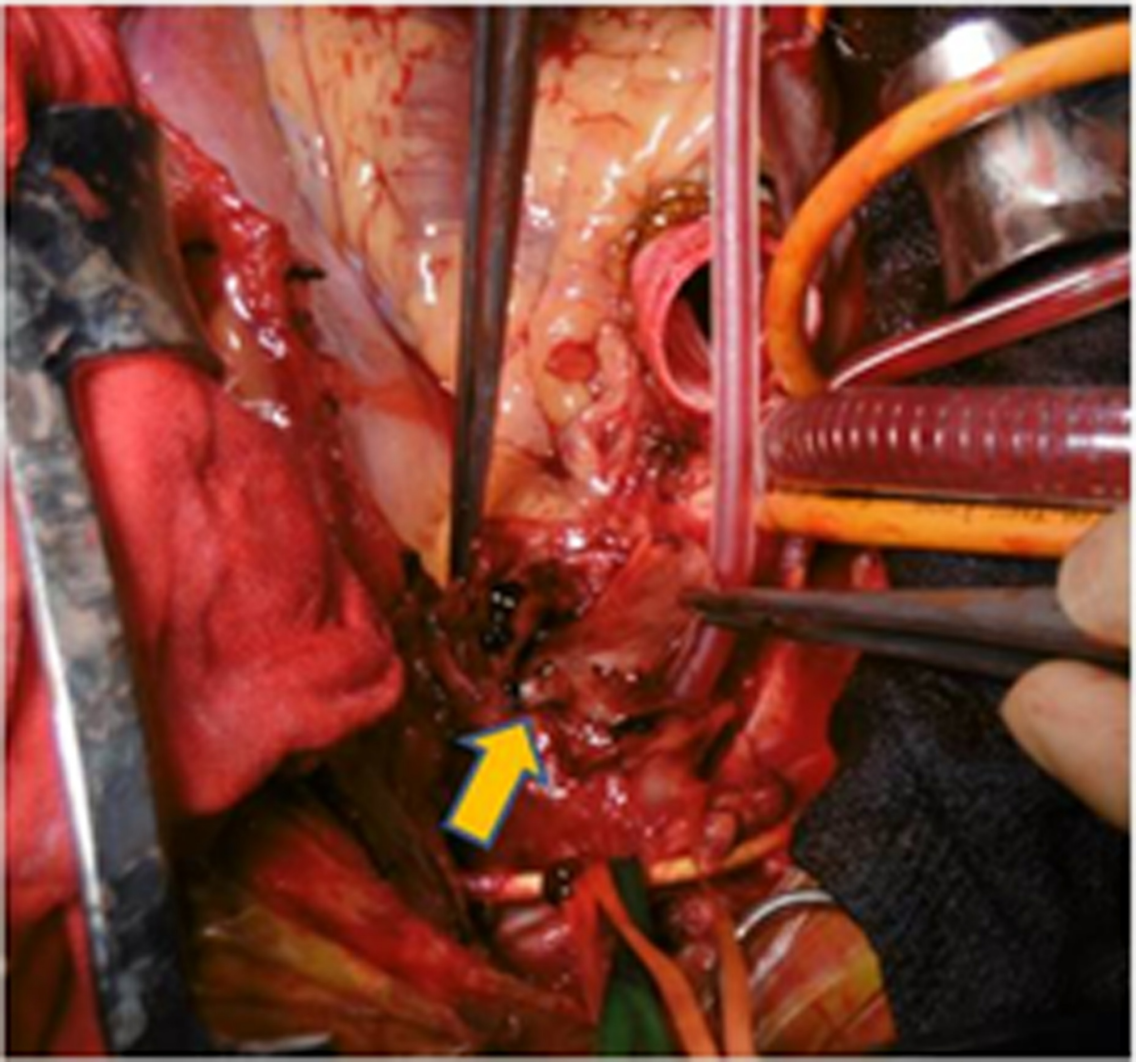
Intraoperative finding: a perforated aortic arch by a bare spring stent at the proximal end of the endovascular stent graft (arrow)

**Figure 5:**
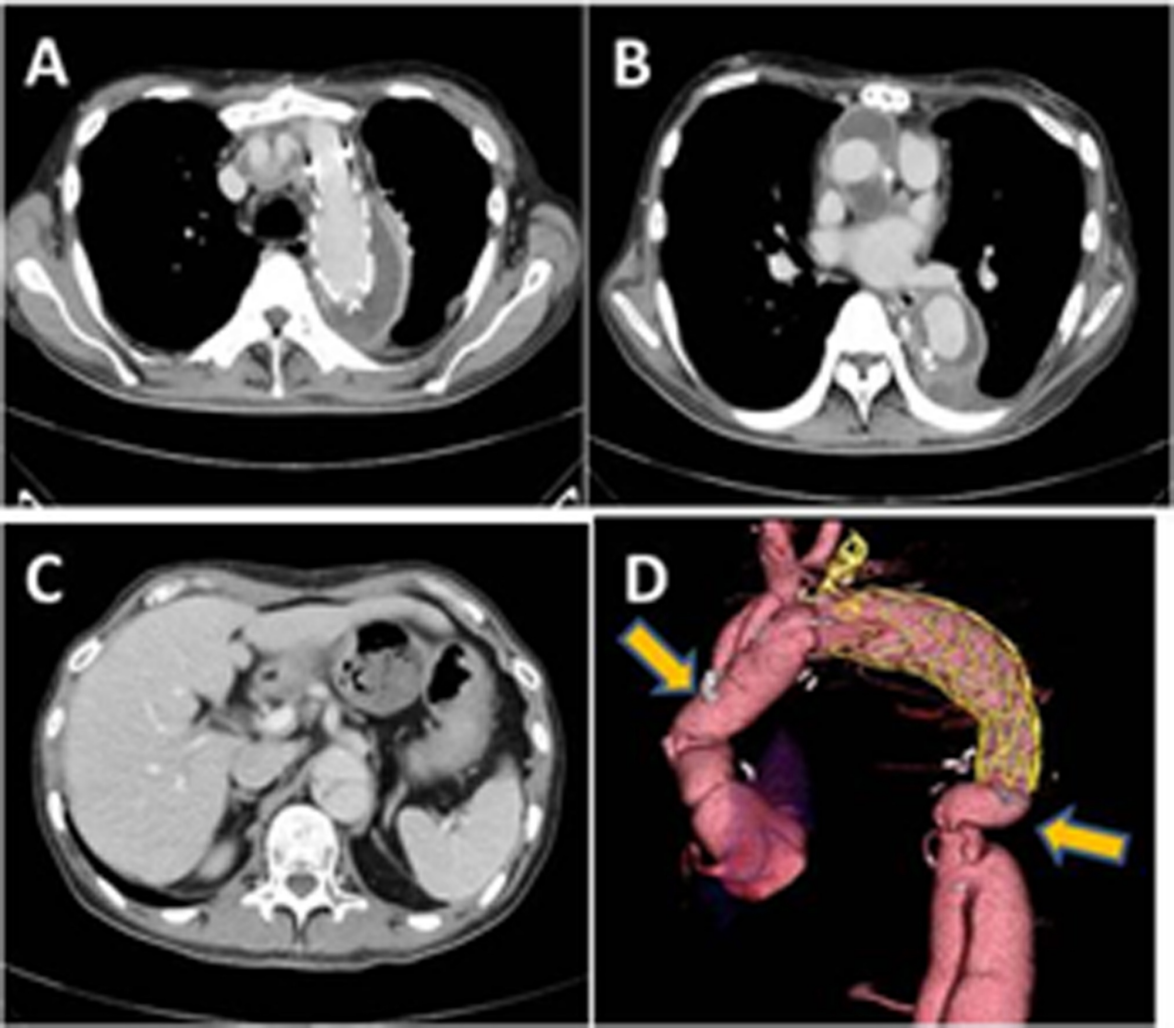
A-D. Chest computed tomography image obtained six months after the operation. No abnormal findings were observed in the replaced ascending and descending aorta (arrow). The false lumen of the descending aorta was completely thrombosed and shrunken.

The patient had an uneventful recovery period. However, seven days after the operation, the patient had sudden chest pain. A CT angiography revealed a forward blood flow in the true lumen of the distal aortic arch (type IA endoleak, Figure 3A) and thrombosed aortic dissection of the ascending aorta (Figure 3B). The maximum diameter of the dissected thoracic aorta exceeded 70 mm. Immediately, total aortic arch and descending aorta replacement were performed under general anesthesia through a median sternotomy with the fifth interspace thoracotomy (T-shape incision and thoracotomy). Arterial cannulation was directly accomplished through the dissected ascending aorta under echo guidance as the orifice of the bilateral subclavian artery were dissected and the TBAD extended to both femoral arteries. And venous cannulation was inserted in the superior and inferior vena cava, right upper pulmonary vein, and coronary sinus. The ascending aorta was clamped after establishing extracorporeal circulation, and myocardial protection was achieved by means of antegrade and retrograde infusion of cardioplegia. After transection of the ascending aorta, a 30-mm Dacron vascular graft (J graft, Japan Lifeline, Tokyo, Japan) was anastomosed to the aortic root. At 25°C, the cross-clamp was removed, and the dissected aorta was opened to the aortic arch till the level of the left subclavian artery. The proximal new entry was seen at the tip of the Variant proximal bare stent (Figure 4). After inspection, the bare waved wire of the endovascular graft proximal edge was cut to facilitate suturing. Endovascular stent grafts at the time of the last operation could not be removed. Bilateral antegrade cerebral perfusion was accomplished through selective cannulation of the brachiocephalic artery and left common carotid artery. The LSA was also perfused through the previous bypass. Before arch replacement, distal descending aorta replacement was performed with a 26-mm Dacron graft immediately distal to the endovascular graft. At the proximal side, the Dacron graft was anastomosed with the stent graft and native descending aorta, and the distal side was anastomosed with a double-barrel technique because the major aortic branches had a false lumen origin. Next, the four-branch Dacron graft was anastomosed just above the level of the left subclavian artery with the deployed endovascular graft. The branched vascular graft was anastomosed to the healthy tissues of the brachiocephalic artery and left common carotid artery. Finally, the proximal and distal graft was anastomosed. The patient was gradually weaned from the cardiopulmonary bypass. The circulatory arrest and total cardiopulmonary bypass times were 140 and 200 minutes, respectively. The patient’s postoperative vital signs were stable, and no neurological adverse events occurred. He was awake and extubated on postoperative Day 2. He was discharged without any complications. At the six-month follow-up, contrast-enhanced CT demonstrated that there was no problem at the anastomotic site and the false lumen at the descending aorta was completely thrombosed and shrunken (Figure 5).

## Discussion

According to international guidelines, stable patients with uncomplicated TBAD should receive optimal medical treatment with aggressive blood pressure control [12]. Rupture, malperfusion, hemothorax, intramural hematoma, refractory pain, and limb ischemia result in complicated TBAD in the acute phase. The results from the International Registry of Acute Aortic Dissection demonstrated that acute TBAD comprises approximately one-third of all aortic dissection cases and that approximately 25% of patients presenting with TBAD have complicated TBAD [13]. Untreated complicated dissection can result in severe organ damage or death. Therefore, efficient and timely interventions should be adopted to save the lives of patients.

Although great advances have been made over the years, the management of complicated TBAD with open surgical techniques is associated with high mortality and morbidity rates (morbidities include spinal cord or visceral ischemia, cerebrovascular accident, and renal failure). Recently, TEVAR for complicated TBAD has been increasingly performed due to its lower morbidity and association with a significantly lower hospital or 30-day mortality (4% to 10.6%) than that seen with conventional open surgical repair (19% to 40%) [1-3]. Nowadays, open surgery is rarely performed in cases of complicated TBAD and has been replaced largely by endovascular therapy. Therefore, TEVAR has been listed as a class I procedure recommended for the treatment of complicated TBAD [12].

Endovascular repair is also associated with significant complications. Aneurysm development, aortic rupture, stroke, paraplegia, bowel infarction, limb ischemia, endoleaks, endoprosthesis migration, aortoesophageal fistula, and RTAD have all been described [2]. RTAD is a fatal complication of TEVAR, with an incidence ranging from 1.3% to 17.9% and a mortality rate ranging from 33.4% to 42.0% [14]. It is an emergency that requires surgical repair and often requires ascending and aortic arch reconstruction with hypothermic circulatory arrest or endografting. The operative strategies for the rescue of these patients from this complication are similar to those for acute type A dissection. The frozen elephant trunk technique is the preferred choice for patients with a dissection tear involving the arch [15]. Endografting for RTAD is one of the most surgically challenging types of aortic dissection because the closure of the primary tear is not easy with endografting [11]. Most patients with RTAD had landing zones in the more proximal aorta. Stent graft devices had barbs, and some patients had proximal bare stents. Therefore, at the time of surgery, it is necessary to cut a part of the stent graft, and it is difficult to remove all stent grafts in many patients.

The occurrence of RTAD following TEVAR is associated with procedure-, device-, and aorta-related events. A rough wire or catheter manipulation might injure the fragile aortic wall, which is considered to be associated with intraoperative or early postoperative RTAD. Additionally, balloon touch-up is considered to be associated with intraoperative RTAD. In our case, intraoperative findings showed that new entry tears were located at the top of the proximal bare spring stent, despite landing proximal to a healthy undissected vascular wall in TEVAR. There was direct evidence of stent graft-induced injury at the surgery in half of the patients analyzed in a multicenter study [14].

The primary goal of TEVAR for TBAD is true lumen expansion and false lumen decompression (or thrombosis). When branch vessels or distal re-entry are supplied by the false lumen, after successful TEVAR in TBAD, the high-pressure gradient of the false lumen is a serious problem. In our first operation, blood flow from the re-entry to the thoracic false lumen was considerably high even on angiography. Therefore, the patent false lumen of the descending aorta by residual entry is likely to require distal reoperation in the longer term and may cause organ malperfusion or rupture in the acute phase [16]. For this reason, we performed total arch and descending aorta replacement and preserved the distal part of the stent graft. The key issue of this procedure is that the arch and descending aorta replacement were performed at the same time for RTAD with the stent graft preserved between them in an acute phase. In addition, in order to prevent the rupture and to avoid the next thoracotomy, we performed a median sternotomy and left 5th interspace thoracotomy (T-shape incision and thoracotomy) because a graft replacement just above the diaphragm was necessary. The distal side of the descending aorta was anastomosed with a double-barrel technique, as many major branches were of false lumen origin. In our case, the patient’s proximal landing zone aortic diameter was approximately 30 mm, and a just-sized endograft was applied (not oversized). The thoracic endograft covered the proximal landing zone by approximately 30 mm. For TBAD, both the length of the landing zone and the diameter of the endograft were mentioned within the instruction for use. There were no complications during the first procedure. We suspected that the aortic intima was slightly damaged by the proximal bare stent, which caused a minor tear during the acute phase. However, in many cases of these dissections, the aortic false lumen remains patent, with potential risks of aneurysmal dilatation and rupture requiring surgical intervention.

In the present case, we planned to perform total arch replacement, remove the bare stent, and perform distal anastomosis to the previous endograft. However, the management of the implanted endograft was highly difficult because it was anchored onto the aortic wall, and there was no easy access to the implanted endograft and dissected descending aorta through median sternotomy. However, as the patient showed no evidence of malperfusion, we considered this surgical repair as effective in this emergency situation. In our case, a follow-up CT scan at six months after the operation showed complete thrombosis and shrinkage of the false lumen of the descending aorta. Moreover, the diameter of the abdominal aorta was not larger than that recorded.

## Conclusions

RTAD is a rare but serious complication that can present early after TEVAR for TBAD because of the strength of proximal bare stents and emergent surgical repair. When RTAD occurs after TEVAR for type B dissection accompanied by a huge descending aortic aneurysm, it is very important to decide whether open or hybrid surgical repair would be the most effective management approach. A combination of total arch and descending aorta replacement while partly preserving the previous stent graft is feasible in terms of surgical repair through a median sternotomy with a left thoracotomy for RTAD after TEVAR. Altogether, for RTAD after TEVAR performed for complex type B dissection with a descending aortic aneurysm, the procedure described herein is safe and effective and can avoid the necessity of acute and mid-term re-interventions.
